# Primary hemochromatosis presented by porphyria cutanea tarda: a case report

**DOI:** 10.4076/1757-1626-2-7246

**Published:** 2009-06-17

**Authors:** H Jorn Bovenschen, Wynand H P M Vissers

**Affiliations:** Department of Dermatology, Radboud University Nijmegen Medical CenterRené Descartes dreef 1, P.O Box 9101, 6525 GL, NijmegenThe Netherlands

## Abstract

We present a 27-year-old female Caucasian patient, who initially presented with extensive fragility and blistering of mainly the dorsal side of both hands. Histology and urine porphyrin analysis confirmed the diagnosis of porphyria cutanea tarda. Internal screening for underlying disease revealed C282Y mutation-associated primary hemochromatosis, a hereditary iron-overload syndrome that may cause toxicity of a variety of organs. Hemochromatosis and porphyria cutanea tarda are pathogenetically linked as iron interferes with heme synthesis pathway. Patient was successfully treated with phlebotomy and low-dose hydroxychloroquine.

## Introduction

Primary hemochromatosis is a hereditary iron-overload syndrome with enhanced intestinal absorption of iron and potentially noxious iron in the peripheral tissue. Two separate gene mutations in the hemochromatosis gene (C282Y and H63D) have recently been identified that are responsible for the disease [1]. However, other inherited, acquired or environmental triggering factors are necessary to develop pathology. Secondary hemochromatosis may be due to alcohol use, excessive iron and vitamin C intake, oral contraceptives and blood transfusions [2,3]. Early recognition of hemochromatosis is essential for preventing destructive organ damage. The initial signs and symptoms are quite diverse, as multiple organs may be affected. We present a patient with sunlight-aggravated fragile skin on the hands and wrists with bullae formation, diagnosed as porphyria cutanea tarda (PCT), which was the first sign of underlying hemochromatosis.

## Case presentation

**Figure 1.**
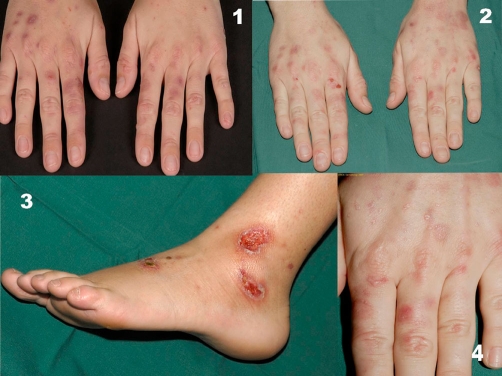
Skin abnormalities in our patient. Erosive erythematous patches, bullae and milia on the dorsal side of both hands (**1, 2, 4**). Ulcerations on the lower left leg caused by beta-hemolytic streptococci group A (**3**).

**Figure 2.**
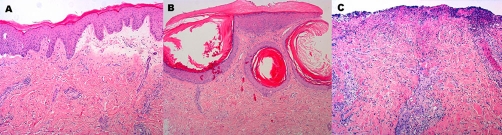
Histopathology of the skin lesions. Histopathology (hematoxylin-eosin staining) of active PCT lesions on the dorsal aspect of the hands (**A**), milia formation (**B**) and the non-specific ecthyma (**C**) developing in our patient. (Magnification 100x)

A 27-year-old female Caucasian patient presented with suddenly appearing blisters and fragility of the extensor sides of both hands since May 2007. The lesions were progressive and not directly sensitive to light. Patient was otherwise healthy and used only an oral contraceptive: Diane-35. There was no atopy. There was no skin disease or similar skin symptoms in family members. There was only modest alcohol use and no smoking. Dermatological examination revealed multiple active blisters, erosions, erythematous macules, dyspigmentation, atrophic scarring and milia on the extensor sides of both hands (Figure 1). Histopathology of lesional and perilesional skin showed a partial normal epidermis with bullae formation at the basal membrane. Herein, fibrin deposition and granulocytes were observed in addition to a perivascular infiltrate and a vasculopathic reaction pattern deep in the dermis (Figure 2). Blood analysis revealed: CRP 34 (n < 10), ASAT 91 (n < 40), ALAT 141 (n < 45), Fe 41 (10-25) and ferritin 783 (6-80). Differential diagnosis at this time included PCT, epidermolysis bullosa acquisita, bullous pemphigoid, perniones, drug eruption. Urine porphyrin screening showed a raised uroporphyrin (1642 nmol/mmol creatinin (n < 2.5)) and heptaporphyrins, in combination with relative normal other porphyrins. There was normal serum protoporphyrin. With the selective abnormal laboratory parameters, normal autoimmune laboratory analysis, negative hepatitis and HIV serology and normal liver ultrasonography, suspicion had been raised of a primary hemochromatosis. Gene analysis ultimately showed a homozygous C282Y missense mutation of the hemochromatosis (HFE) gene, in the absence of a H63D mutation.

The final diagnosis was porphyria cutanea tarda due to primary hemochromatosis and the course was as follows. Patient was instructed to stop using alcohol, which she already sparingly used. She was advised to stop using her contraceptive medication as well. The patient was repetitively treated with phlebotomy which resulted in a decrease in ferritin levels (783 to 100) in three months time. Adjuvant low-dose hydroxychloroquine (200 mg daily) was started afterwards to prevent the possibility of a relapse. Four months later, the porphyria symptoms were still in remission. However, she rapidly developed three ulcers on the lateral side of the left lower leg in 5 days varying from 6 mm to 2 cm in diameter (Figure 1). Histology was consistent with ecthyma and not with vasculitis or venous ulceration (Figure 2). Bacterial cultures revealed massive growth of beta-hemolytic Streptococci Group A and Staphylococcus aureus. With a diagnosis of ecthyma patient was treated with local mupirocin (Bactroban®) ointment and compression (Tubigrips®). Clearance of the infection was reached in 10 days.

## Discussion

In our patient, fragile skin and bullae formation on the dorsal side of the hands related to PCT is the presenting symptom of underlying primary hemochromatosis. Hemochromatosis is the most common inherited liver disease and the most common autosomal recessive genetic disorder [4]. From epidemiological point of view there is a clearcut association between PCT and hemochromatosis [5]. The question rises how porphyria, hemochromatosis and/or the hemolytic streptococcal infection can be linked together.

Most patients with heriditary hemochromatosis have a mutation in one of the mentioned HFE genes located on chromosome 6 [6]. Hemochromatosis interferes with the transferrin receptor and causes a clear decrease in the affinity with which the receptor binds transferrin. This interaction also may modulate cellular iron uptake and decrease ferritin levels. When a mutant or nonfunctional variant of the HFE gene is present, ferritin levels are not under influence of a normal and functional HFE gene, which leads to enhanced accumulation of iron in peripheral tissues [7].

**Figure 3.**
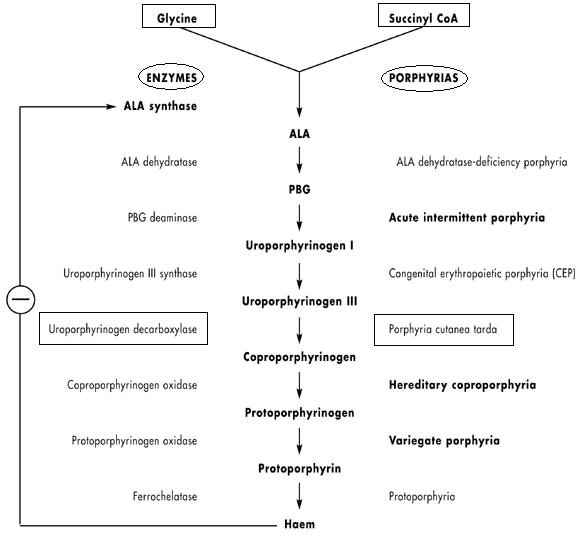
Heme synthesis, enzymes and porphyrias.

The term “porphyria cutanea tarda” originally described the dermatological manifestations of various chronic porphyrias. Its usage now is usually restricted to disorders associated with a deficiency of uroporphyrinogen decarboxylase (UROD). It is UROD that is responsible for an essential step in the heme synthesis pathway: the conversion of uroporhyrinogen III into coproporphyrinogen III [7]. This is reflected in Figure 3.

Iron overload and in particular the cellular modifications of the iron status secondary to hemochromatosis mutations affect the quantity and activity of UROD [8]. Iron, by catalyzing the formation of reactive oxygen species, can enhance uroporphyrin formation by increasing the rate at which uroporphyrinogen is oxidized to uroporphyrin. Iron may also act indirectly to inhibit UROD activity by enhancing the formation of non-porphyrin products of porphyrinogen oxidation that are themselves direct inhibitors of the enzyme. Finally, iron can act to increase urophorphyrin production by inducing δ-aminolevulinic acid synthase, the precursor to uroporphyrinogen, inside the cell [4]. With the deficiency in UROD activity heme precursor uroporphyrinogen accumulates in the skin. This molecule is highly photosensitive, causing blistering and fragility on the UV-exposed skin and subsequent complement activation and histamine release. Since a considerable amount of iron is required to produce symptoms, this type of PCT most often starts in the third decade of life. Secondary causes contributing to the disease, such as alcohol use, excessive iron and vitamin C intake, oral contraceptives and blood transfusions, are often observed and should also be eliminated. From epidemiological point of view PCT and hemochromatosis are also heavily linked together.

The ecthyma caused by group A beta-hemolytic streptococci our patient developed could not be explained by either hemochromatosis or PCT. One report highlights the increased risk of venous leg ulceration in patients with the C282Y mutation [9]. However, given the rapid development of three ulcers, the bacterial cultures that revealed beta-hemolytic streptococci group A and a rapid response to local treatment, a venous origin of the leg ulcerations in this young patient is not likely.

Treatment of PCT is based on recurrent phlebotomies and low-dose (hydroxy)chloroquine. Our patient had an excellent response to these treatments [2,4,6]. However, there is evidence that patients that are homozygotes for the C282Y mutation suboptimally respond to (hydroxy)chloroquine therapy in contrast to their heterozygote and wild-type counterparts [6].

## Conclusion

In conclusion, PCT may be the presenting symptom of underlying primary hemochromatosis. Urine analysis is necessary to confirm the diagnosis of PCT. DNA analysis may reveal HFE mutations, where iron and ferritin values in peripheral blood are relevant screening parameters for hemochromatosis activity. Treatment consists of removing probable secondary disease aggravating factors, photo protection, recurrent phlebotomies and low-dose hydroxychloroquine.

## List of abbreviations

HFE: hemochromatosis
PCT: Porphyria cutanea tarda
URDO: Uroporphyrinogen decarboxylase
